# Ozone–Oxygen Therapy to Prevent HPV-Related Cancers of the Lower Gynecological Tract in Infected Patients: The Rationale for Further Developments

**DOI:** 10.3390/cancers17030543

**Published:** 2025-02-06

**Authors:** Luca Roncati

**Affiliations:** Department of Life Sciences, Health, and Health Care Professions, Link Campus University, 00165 Rome, Italy; l.roncati@unilink.it or emailmedical@gmail.com

**Keywords:** ozone, ozone–oxygen therapy, vaginal insufflation, human papillomavirus (HPV), HPV infection, precancerous lesions, cancer, lower gynecological tract, cervix uteri, vagina

## Abstract

Background: O_3_-O_2_ therapy is an alternative medical treatment that introduces a mixture of O_3_-O_2_ into the body for therapeutic purposes. The objective of this study is to evaluate its margins of applicability in the eradication of HPV infection from the lower gynecological tract by means of vaginal insufflation. Methods: An in-depth review of the international literature on this topic is carried out; in addition, O_3_-O_2_ therapy is compared with other treatments currently available in terms of its advantages, disadvantages, and exploited technologies. Results: The possible benefits and limitations of O_3_-O_2_ vaginal insufflation are explained in detail; overall, it appears to be an interesting tool as part of complex management to prevent HPV-related cancers of the lower gynecological tract in infected patients. Conclusions: The rationale and guidelines of this innovative procedure have been successfully illustrated, providing the technical specifications for further developments.

## Simple Summary

The human papillomavirus (HPV) is the leading cause of infection-attributable cancers in women worldwide; among these, cervical cancer is the most common gynecological tumor and the second most frequent female-specific malignancy after breast cancer. Since virtually all carcinomas of the lower gynecological tract are due to high-risk HPV, new prevention strategies based on HPV eradication in infected patients are needed. Thanks to its well-known antiseptic properties, ozone–oxygen (O_3_-O_2_) therapy appears to be an interesting tool in order to achieve this goal by means of vaginal insufflation. Here, the rationale and guidelines for this prospective procedure are illustrated in detail.

## 1. Human Papillomavirus

The human papillomavirus (HPV) is a double-stranded circular DNA oncovirus belonging to the *Papillomaviridae* family; today, more than 200 different genotypes are known [1]. Its oncogenic power is due to the early (E) expression of E6 and E7 proteins (p), which are able to inactivate the tumor suppressors p53 and retinoblastoma, respectively [2]. This inactivation may be accompanied by cytoskeleton alterations of the infected cells, a phenomenon called koilocytosis, and it varies from genotype to genotype. Therefore, high-risk HPV (16, 18, 31, 33, 35, 39, 45, 51, 52, 56, 58, 59, 68, 73, 82) and probable high-risk HPV (26, 53, 66) are distinct from low-risk HPV (6, 11, 40, 42, 43, 44, 54, 61, 70, 72, 81, 89), where risk means the probability of neoplastic transformation [3].

Since the main route of virus transmission is sexual intercourse, the most frequent sites of HPV-related cancers are the cervix, vagina, vulva, anus, penis, and oropharynx. Before becoming invasive, they present with grade 1 (mild), grade 2 (moderate), and grade 3 (severe) dysplastic changes; to describe these progressions, which usually take years, we refer to acronyms such as CIN (cervical intraepithelial neoplasia) 1/2/3, VaIN (vaginal intraepithelial neoplasia) 1/2/3, VIN (vulvar intraepithelial neoplasia) 1/2/3, AIN (anal intraepithelial neoplasia) 1/2/3, and PeIN (penile intraepithelial neoplasia) 1/2/3.

**Figure 1:**
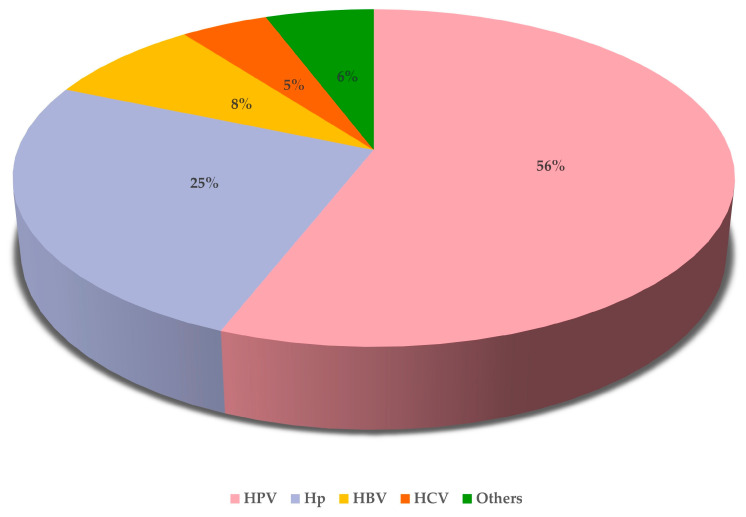
Percentage of distribution by infectious agents of global cancer cases among females attributable to infections in 2020 (total attributable cases: 1,200,000.00) with a focus on the four most prevalent agents, i.e., HPV, Hp (Helicobacter pylori), HBV (hepatitis B-virus), and HCV (hepatitis C-virus) [data source: Globocan].

According to the Global Cancer Observatory (Globocan) provided by the International Agency for Research on Cancer of the World Health Organization, in 2020, the global number of cancer cases among women attributable to infections was 1,200,000.00 [4], of which 56% was caused by HPV, which, therefore, represents the main oncogenic infectious agent for the female population in the world (Figure 1).

## 2. Cervical and Vaginal Cancer

Cervical cancer is the most common tumor of the gynecological tract and the second most frequent female-specific malignancy after breast cancer; suffice it to note that in 2022, there were 662,301 new cases and 348,874 related deaths worldwide [5].

The continent with the highest rates of incidence and mortality was Asia (60.0% vs. 57.3%), followed by Africa (19.0% vs. 23.1%), Latin America and the Caribbean (9.5% vs. 9.6%), Europe (8.8% vs. 7.7%), Northern America (2.4% vs. 1.9%), and Oceania (0.37% vs. 0.38%) [5]. From these data emerged the implementation of mass screening programs, which has significantly reduced both incidence and mortality in developed countries.

As virtually all cervical carcinomas are caused by high-risk HPV, in particular strains 16 and 18, modern screening programs include HPV testing alongside the traditional Pap smear. The latter allows us to identify precancerous lesions of both squamous and glandular epithelium, which can still be handled with conservative approaches, such as conization.

Contrariwise, vaginal cancer is the rarest, with 18,819 new cases and 8240 related deaths around the world in 2022 [6]; because it is so rare, no screening programs have been implemented. Asia remains the continent with the highest rates of incidence and mortality (54.0% vs. 56.7%), followed by Europe (16.8% vs. 16.3%), Africa (10.9% vs. 13.6%), Northern America (8.8% vs. 7.0%), Latin America and the Caribbean (8.6% vs. 5.7%), and Oceania (0.79% vs. 0.73%) [6]. Vaginal cancer usually occurs in the upper third of the vagina, and the squamous histotype is strongly associated with HPV infection, similar to cervical carcinoma.

## 3. Ozone–Oxygen Therapy

Ozone–oxygen (O_3_-O_2_) therapy is an alternative medical treatment that introduces a mixture of O_3_-O_2_ into the body for therapeutic purposes [7,8]. O_3_ is one of the most powerful oxidizing agents in nature, known to react directly with organic compounds; for this reason, it is produced by neutrophils in the biochemical process of destroying pathogens [9]. When O_3_ breaks down into O_2_, it gives rise to oxygen-free radicals, which belong to highly reactive oxygen species and can further damage organic molecules [10,11]; moreover, tissue oxidation triggered by O_3_ is thought to contribute to inflammation and clearance [11]. The inactivation and killing of viruses are the result of peroxidation reactions between O_3_ and the biomolecules that constitute their essential structures, i.e., the nucleocapsid and, for enveloped viruses, the envelope [11,12].

In 1892, the journal *The Lancet* published an article describing the effectiveness of administering ozonated water for the treatment of tuberculosis, and four years later, Nikola Tesla manufactured the first O_3_ generator [11,13]. Since then, O_3_-O_2_ therapy has been tested on humans for the treatment of various viral diseases such as hepatitis B and C [14,15], herpes zoster [16,17,18], Ebola [19], HIV/AIDS [20,21,22,23], and SARS-CoV-2/COVID-19 [24,25,26,27,28,29,30,31,32,33,34,35]. Through an experimental study on transgenic female mice, Portuguese researchers have recently demonstrated that O_3_ is also effective against lesions induced by HPV16; in fact, 85.7% of untreated transgenic mice have shown dysplastic lesions, compared to only 28.6% of O_3_-treated mice [36].

Moreover, its virucide, bactericide, and fungicide properties are currently exploited in the treatment of wounds, burns, and skin infections, including warts, by means of ozonide gel and creams or by intralesional delivery [37,38,39,40,41]. A further successful field of O_3_ application is in dentistry to eradicate the bacteria responsible for caries, where gas is conveyed directly onto the decayed tooth, sealed by a small cup connected to the O_3_ generator via a silicone tube [42,43,44].

## 4. Vaginal Insufflation

In medicine, insufflation means the introduction of gas into a hollow organ, just like the vagina; vaginal insufflation is, therefore, one of the possible systemic routes of O_3_ administration, and maximum caution is required to ensure patient safety [45]. The rationale as to why the O_3_-O_2_ mixture can be used for vaginal sanitation is based on the well-known antiseptic properties of O_3_; in this regard, the main vaginal pathogens such as *Candida albicans*, *Gardnerella vaginalis*, *Trichomonas vaginalis*, and *Chlamydia trachomatis* are not resistant to O_3_ exposure [46,47,48,49,50,51]. For personal hygiene, O_3_ washes and ovules have been available on the market for some time.

While conization represents the treatment of choice for high-grade squamous intraepithelial lesions (HSILs), i.e., the cytological counterpart of the histological categories CIN2/3, and atypical glandular cells (AGCs) favor neoplastic disease, both confirmed by colposcopy-guided biopsy, no consensus has been reached on how to treat high-risk HPV+ low-grade squamous intraepithelial lesions (LSILs), i.e., the cytological equivalent of CIN1 HPV+. There are those who opt for follow-up with the regular repetition of Pap/HPV tests, who immediately opt for diathermocoagulation, cryotherapy, laser vaporization, or topical imiquimod (Table 1), and those who propose the anti-HPV vaccination in combination with one of the previous options [52]. Indeed, the anti-HPV vaccination has been shown to be effective not only in preventing precancerous lesions and cervical cancer but also in preventing CIN2/3 recurrence after conization [53]. Similarly, the management of high-risk HPV+ VaIN1/2/3 represents a grey zone, where being as conservative as possible is required. Recent findings suggest that women with high-risk HPV infection and a mild abnormal Pap smear may benefit from nonsurgical therapies [54]. Faced with this scenario, O_3_-O_2_ therapy can find its field of application in the attempt to eradicate infection via the intracavitary route and, consequently, the main risk factor for neoplastic transformation in infected patients. The noteworthy advantages of O_3_-O_2_ therapy compared to the above-mentioned treatments lie in the simultaneous diffusion of the gas into the exocervix, endocervix, and upper third of the vagina, thus allowing the potential eradication of the virus on the entire mucosal surface at greatest neoplastic risk, and this is not limited to the application site as is the case with other treatments (Table 1), in addition to its immunomodulatory effect and concomitant tissue rejuvenation without induced abrasions or ulcerations [55,56,57].

The International Scientific Committee of O_3_ Therapy (ISCO_3_), the independent authority that drafted the “Madrid Declaration on O_3_ Therapy”, which is a reference source for all O_3_ therapists, has provided guidelines and a protocol for vaginal insufflation [58]. Preliminarily, the patient must be informed about the procedure and sign an informed consent form. Exclusion criteria for this treatment are pregnancy and purperium, menstrual bleeding, and favism. Due to an X-linked recessive defect involving glucose-6-phosphate dehydrogenase (G6PD), favism is the most common enzyme-deficiency anemia worldwide, and its prevalence varies among ethnic groups with overall lower frequency in the Pacific (2.9%), Europe (3.9%), and the Americas (3.4%) if compared to sub-Saharan Africa (7.5%), the Middle East (6.0%), and Asia (4.7%) [59]. A G6PD test should be performed prior to O_3_-O_2_ therapy to rule out favism, in addition to a blood assay for human chorionic gonadotropin. In fact, during pregnancy and puerperium, a remote risk of air embolism exists due to non-collapsible veins at the placental site [60]. Relative contraindications and special situations requiring extreme caution are represented by anatomical abnormalities, cardiovascular instability, coagulation disorders, convulsive states, uncompensated hyperthyroidism, alcohol intoxication, substance abuse, Wilson’s disease, hemochromatosis, and copper or iron supplements [58]. In all these circumstances, the procedure is not recommended.

**Figure 2:**
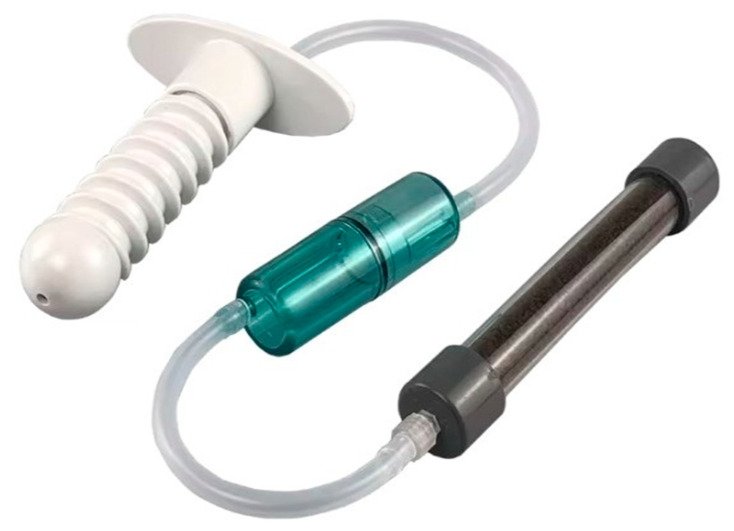
An O_3_-O_2_ therapy probe for vaginal insufflation: the central input of the vaginal insert (white) must be connected to a medical O_3_ generator, while the lateral output of the insert is connected in series by means of a silicon tube with a liquid collector (green) and an O_3_ destructor (black) to avoid gas leaks in the room. The concentric multi-ring thread of the vaginal insert is also designed to prevent O_3_ leakage during treatment.

Once consent has been obtained, vaginal irrigation with saline solution is performed in order to remove any accumulation of mucus. Then, a lubricated probe made of O_3_-resistant material that does not release phthalates is inserted into the vagina; the base of the insert is equipped with two couplings, one central and one lateral, to which two silicone cannulas are connected. The opposite end of the central cannula is connected to an O_3_ generator, while the lateral tube is connected to an O_3_ destructor (Figure 2).

After having placed the patient in the lithotomy position under the control of a pulse oximeter to monitor vital signs, the O_3_-O_2_ mixture is insufflated at a concentration between 10 and 30 μg/mL with constant flow for 5–10 min daily or every other day (Table 2). The volume of the O_3_-O_2_ mixture to be used varies between 1 and 2 L; in total, ten procedures are advised once a year or more, depending on the clinical and laboratory picture [58]. Prolonged concentrations above 30 μg/mL should be avoided due to the increased risk of suppression of the saprophytic flora (Döderlein’s lactobacillus) and to avoid excessive local oxidative stress [58]. The operator must wear glasses, gloves, and a carbon mask as self-protection measures.

The procedure must be immediately stopped in the case of an alteration in the patient’s vital parameters (<90% SpO_2_; >100 bpm). Since O_3_ causes vaginal dryness, it is recommended to lubricate the vagina immediately after the procedure with synthetic mucus made from sodium hyaluronate and to restore the lactobacillary flora with vaginal ovules to be applied in the evening before bedtime.

## 5. Conclusions

O_3_-O_2_ therapy appears to be an interesting tool as part of complex management to prevent HPV-related cancers of the lower gynecological tract in infected patients. HPV testing is necessary to establish an accurate diagnosis and to allow comparisons between the patient’s status before and after O_3_-O_2_ therapy. Further research studies in this direction are, therefore, recommended.

## Figures and Tables

**Table 1:** Advantages, disadvantages, and exploited technologies of existing treatments for HPV eradication from the lower gynecological tract in infected patients compared to O_3_-O_2_ therapy.

Treatment	Tech	Advantages	Disadvantages
**Topical imiquimod**	gel	easy availability easy to use	slides off from the application site mucosal irritation
**Diathermy coagulation**	heat	easy availability well established	action on the application site only mucosal abrasion/ulceration
**N_2_ liquid cryotherapy**	cold	easy availability well established	action on the application site only mucosal abrasion/ulceration
**CO_2_ laser vaporization**	laser	high power focal application	action on the application site only risk of deep penetration
**O_3_-O_2_ therapy**	gas	high diffusion tissue rejuvenation	mucosal irritation remote risk of air embolism

**Table 2:** O_3_ concentrations (μg/mL) achieved by a three-switch O_3_ generator in relation to varying O_2_ flows (L/min): note that the higher O_3_ concentrations are achieved with the lower O_2_ flow. A three-switch generator allows a wider range of achievable O_3_ concentrations than single- or dual-switch devices, depending on the needs and experience of the operator.

O_2_ Flow (L/min)	O_3_ Concentration (μg/mL)		
	Switch I	Switch II	Switch III
1/32	103	104	103
1/16	91	88	83
1/8	80	74	68
1/4	47	43	39
1/2	25	22	20
3/4	16	14	12
1/1	13	12	10

## Data Availability

Data sharing is not applicable to this article.
